# Acute esophageal necrosis complicated by refractory stricture formation

**DOI:** 10.1002/jgh3.12520

**Published:** 2021-03-01

**Authors:** Sunny Sandhu, Timothy Wang, Devang Prajapati

**Affiliations:** ^1^ Department of Internal Medicine University of California Fresno California USA; ^2^ Department of Gastroenterology and Hepatology University of California Fresno California USA; ^3^ Department of Gastroenterology and Hepatology VA Central California Healthcare System Fresno California USA

**Keywords:** acute esophageal necrosis, dilation, esophageal stricture, endoscopy: upper GI < gastroenterology

## Abstract

Acute esophageal necrosis (AEN) is a rare presentation of severe esophageal injury. The optimal long‐term management of complications related to AEN, particularly stricture formation, are not well defined. We report a case of AEN in a patient who presented with diabetic ketoacidosis (DKA) and had dysphagia due to refractory stricture formation after mucosal healing occurred. A 62‐year‐old male with diabetes mellitus presented with altered mental status. He was admitted for hypovolemic shock secondary to DKA and treated with vasopressors, fluid resuscitation, and insulin. After resolution of DKA, he reported persistent dysphagia. Upper endoscopy showed circumferential black mucosal discoloration throughout the entire esophagus that spared the gastroesophageal junction. He was diagnosed with AEN and was continued on a proton pump inhibitor and sucralfate with improvement in symptoms. Repeat endoscopy 4 weeks later showed a 10‐cm benign‐appearing stricture in the mid esophagus. He underwent dilation with temporary symptomatic relief; however, recurrence in symptoms has thus far necessitated a total of 10 repeat upper endoscopies, including repeat dilations along with local steroid injection therapy. AEN is a rare presentation of severe esophageal injury and is typically associated with severe hemodynamic compromise. Although most cases resolve with supportive care and mucosal healing, there is little information regarding prognosis and optimal management of complications, such as refractory esophageal strictures. We describe a case of AEN complicated by refractory symptomatic esophageal stricture despite several dilations and intralesional steroid injections and discuss our approach to treatment.

## Graphical abstract

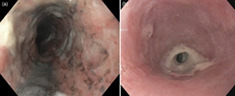

Acute esophageal necrosis (AEN) is a rare presentation of severe esophageal injury. The optimal long‐term management of complications related to AEN, particularly stricture formation, are not well defined. We report a case of AEN in a patient who presented with diabetic ketoacidosis who had dysphagia due to refractory stricture formation after mucosal healing occurred, and discuss our approach to treatment.

## Introduction

Acute esophageal necrosis (AEN), also known as “black esophagus,” is a rare presentation of severe esophageal injury. Initially described postmortem in 1967 by Brennan^1^ and then endoscopically in 1990 by Goldenberg *et al*.,^2^ most cases today are described in isolated case reports. Mucosal injury in AEN results predominantly from a combination of ischemic injury and gastroesophageal acid reflux‐mediated mucosal damage. Most cases occur in patients with underlying cardiovascular comorbidities and alcohol use and are typically associated with cases of severe hemodynamic instability and low‐flow states.^3^ Presenting symptoms can include hematemesis, melena, nausea, vomiting, abdominal pain, dysphagia, or odynophagia.^4^ Hallmark endoscopic findings are a circumferential black mucosal discoloration that abruptly stops at the gastroesophageal junction. AEN carries a mortality rate of 23–35% and can be complicated by superimposed infection, esophageal rupture, and stricture formation.^4^ The optimal long‐term management of complications related to AEN, particularly stricture formation, have not been well defined. We report a case of black esophagus in a patient who presented with severe hemodynamic compromise due to diabetic ketoacidosis (DKA) and had dysphagia due to recurrent stricture formation after mucosal healing occurred.

## Case Report

A 62‐year‐old male with poorly controlled diabetes mellitus with multiple prior admissions for DKA, hypertension, hyperlipidemia, and tobacco use presented to the hospital with altered mental status after being found down confused on the floor of his home after 4 days of last seen normal. He was reported to have an episode of coffee‐ground emesis prior to presentation. Vitals on presentation were notable for hypotension with a blood pressure of 82/47 mmHg. Exam showed dry oral mucosa and altered mental status. Labs were significant for leukocytosis and multiple metabolic derangements, including hyponatremia, hyperkalemia, metabolic acidosis, acute renal failure, and hyperglycemia with a blood glucose of 1878 mg/dL. He was admitted for hypovolemic shock secondary to DKA and was treated with vasopressors briefly, fluid resuscitation, and insulin.

**Figure 1:**
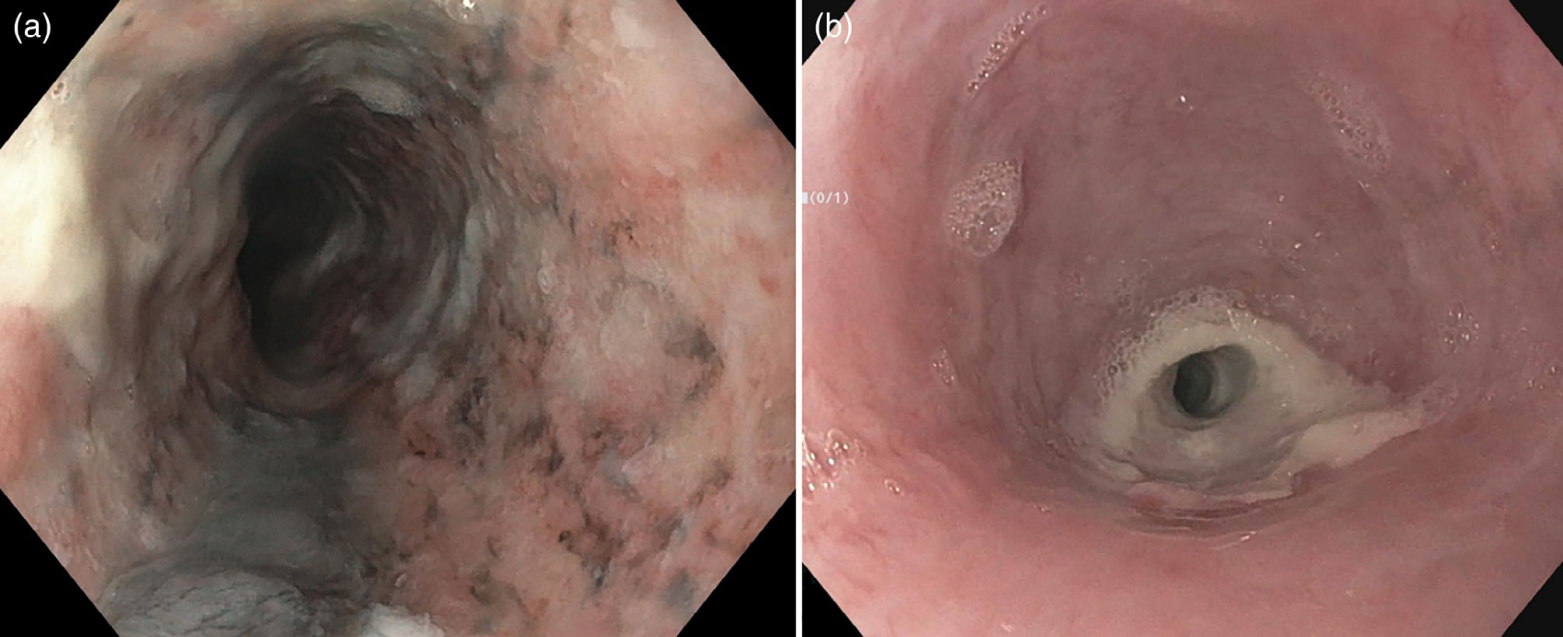
(a) Endoscopic evidence of ‘black’ mucosa with associated esophagitis. (b) Repeat endoscopy at 4 weeks with severe stricture in mid‐esophagus.

After resolution of DKA, he reported persistent dysphagia, epigastric pain, nausea, coffee‐ground emesis, and melena. The gastroenterology department was consulted on day 10 of admission, and upper endoscopy was performed, which showed friable circumferential black mucosal discoloration throughout the entire esophagus that spared the gastroesophageal junction (Fig. 1a). He was diagnosed with AEN and continued on a proton pump inhibitor and sucralfate with improvement in symptoms. Repeat upper endoscopy was performed 4 weeks after discharge, at which time the patient was endorsing recurrent dysphagia, epigastric pain, and nausea. Upper endoscopy revealed a severe benign‐appearing stricture 10 cm in length in the mid‐esophagus that could not be traversed with the endoscope. The stricture was dilated up to 33Fr (11 mm) using a Savary dilator over a guidewire. Dilation led to temporary symptomatic relief; however, recurrence in symptoms has thus far necessitated a total of 10 repeat upper endoscopies, including repeat dilations along with local steroid injection therapy with 80 mg of triamcinolone acetonide split into four‐quadrant injections (Fig. 1b, Figures S1–S5, Supporting information). Dilation intervals were increased from every 2 weeks initially to every 4–8 weeks given symptomatic improvement with sequential dilations.

## Discussion

AEN is a rare clinical entity, with an estimated incidence of about 0.01%. Our patient developed AEN likely due to profound hemodynamic compromise in the setting of hypovolemic shock due to DKA. DKA is known to be one of the most common etiologies of AEN.^5^ The associated chronic atherosclerotic changes that can result in diminished baseline perfusion and the acute hypoperfusion related to the low‐flow state in the setting of osmotic diuresis are thought to be factors that predispose diabetic patients to AEN.^3^ Due to its relatively limited blood supply, the distal esophagus is preferentially affected, while findings of panesophageal involvement is less common. A weakened local mucosal defense system to chemical reflux‐mediated damage due to decreased acid buffering in the setting of critical illness is also thought to play a role.^4^ Finally, general debility and poor nutritional status, which are much more common in patients with multiple comorbidities, are known associated factors.^3^

While data are limited, the mainstay of therapy involves identifying and treating the underlying disease and supportive care to allow for mucosal healing as most patients will achieve endoscopic resolution within several days.^4^ Acute complications such as esophageal rupture are typically correctable if recognized and resolved early. However, long‐term complications such as stricture formation can lead to significant ongoing symptoms. Fibrosis of esophageal mucosa is a known protective mechanism triggered by prolonged inflammation and injury.^6^ The incidence of esophageal stenosis or stricture formation has been described in about 10% of cases of AEN. Discontinuation of injurious agents such as non‐steroidal anti‐inflammatory drugs (NSAIDS), and treatment with gastric acid suppressive therapy and endoscopic dilation if necessary typically result in complete resolution.^4^ This supports the role of early endoscopy for timely diagnosis and prompt treatment.^7^ However, data regarding more severe or refractory cases of this particular complication and optimal long‐term management is extremely limited. In general, refractory benign esophageal strictures can be a therapeutic challenge, with options ranging from repeat dilations and intralesional steroid injections to stent placement and surgical repair.^8^ The majority of benign esophageal strictures are typically managed in a stepwise manner using various nonsurgical means. In fact, more than 90% of benign esophageal strictures are treated successfully with endoscopic dilation.^9^ Surgical resection can be considered only rarely in recalcitrant cases, which have definitively failed nonoperative management with medical and endoscopic therapies. Implications of surgical repair are not only limited to immediate surgical complications but can also include recurrent stricture formation at the anastomotic site.^10^

Although our patient was started on early proton pump inhibitor therapy and sucralfate and had several endoscopic dilations performed, he continued to have recurrent symptoms necessitating repeat dilations. We postulate that this may be a consequence of more severe injury, which was represented by panesophageal involvement noted on index endoscopy. This underscores the importance of prompt diagnosis and treatment of AEN. Further long‐term follow up of patients with severe AEN presentations, particularly those complicated by esophageal stricture, is needed to better understand the disease course, with the goal of anticipating and preventing these long‐term complications, as well as optimizing the approaches to chronic management.

Patient informed consent was obtained for case publication.

## Supporting information


**Figure S1.** Endoscopic evidence of ‘black’ mucosa with associated esophagitis.
**Figure S2.** Endoscopic image showing mucosal sparing of the GE junction.
**Figure S3.** Repeat endoscopy at 4 weeks with severe stricture in mid‐esophagus.
**Figure S4.** Endoscopy at 4 months with persistent stricture.
**Figure S5.** Endoscopy at 6 months with evidence of esophagitis and nodular‐appearing esophageal mucosa.
