# Supplementary material for: Acute esophageal necrosis complicated by refractory stricture formation
Source: JGH Open. 2021 Mar 1;5(4):528–30. doi: 10.1002/jgh3.12520 (PMC8035479; doi:10.1002/jgh3.12520)
Supplement: Supplementary file 1 — Figure S1. Endoscopic evidence of ‘black’ mucosa with associated esophagitis. Figure S2. Endoscopic image showing mucosal sparing of the GE junction. Figure S3. Repeat endoscopy at 4 weeks with severe stricture in mid‐esophagus. Figure S4. Endoscopy at 4 months with persistent stricture. Figure S5. Endoscopy at 6 months with evidence of esophagitis and nodular‐appearing esophageal mucosa. [file JGH3-5-528-s001.docx]

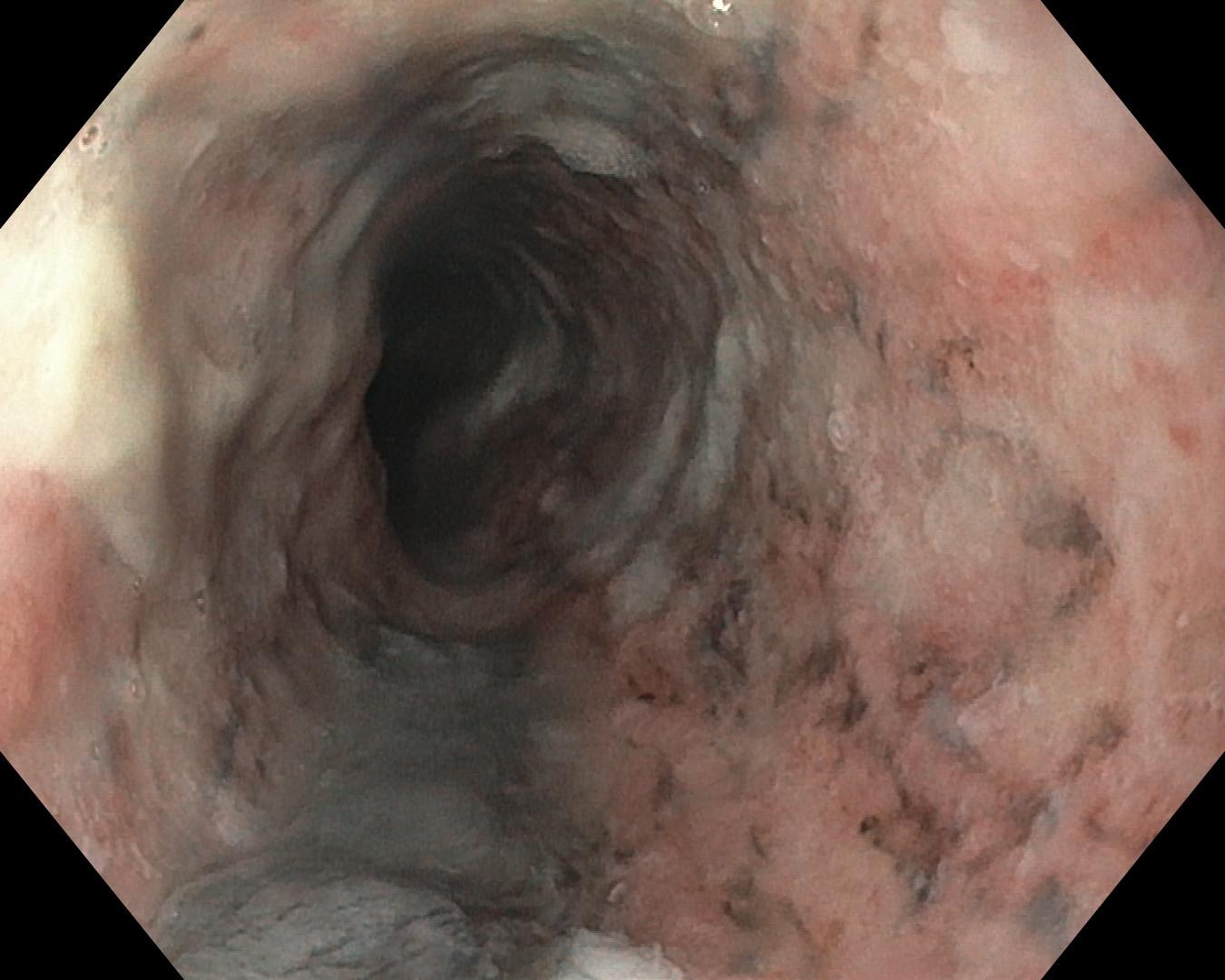
**Supplementary Figure 1.** Endoscopic evidence of ‘black’ mucosa with associated esophagitis.
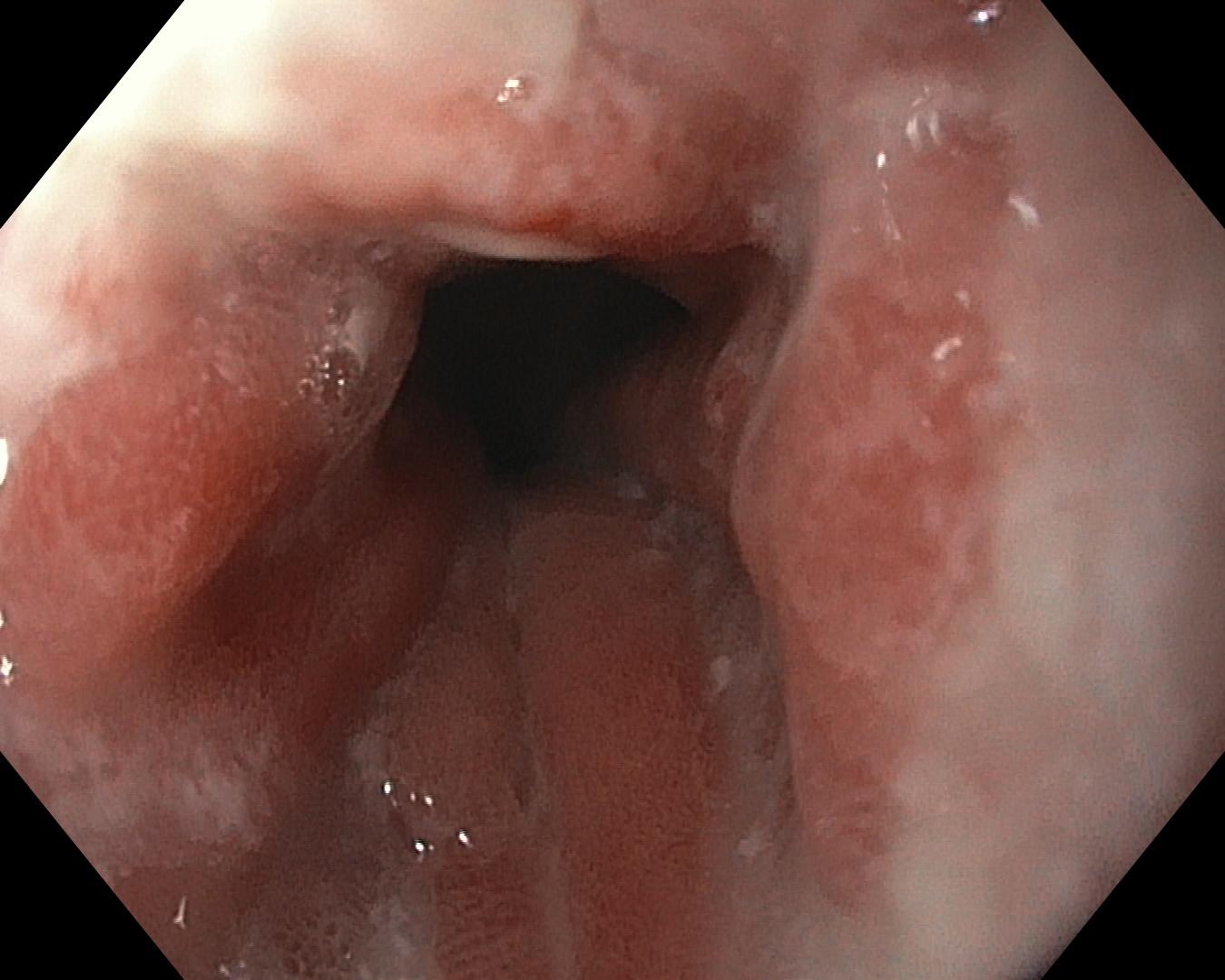


**Supplementary Figure 2.** Endoscopic image showing mucosal sparing of the GE junction.


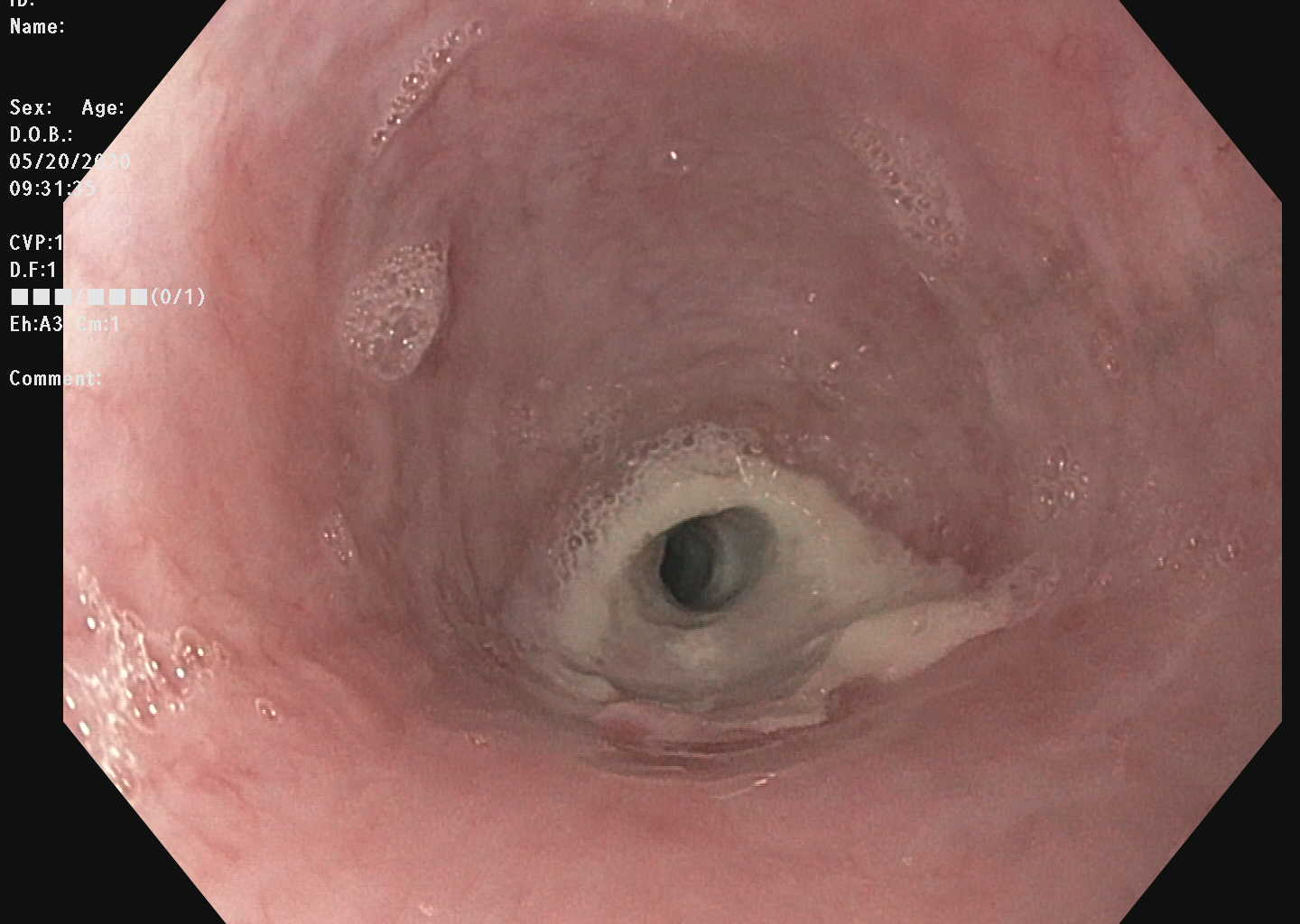


**Supplementary Figure 3.** Repeat endoscopy at 4 weeks with severe stricture in mid-esophagus.


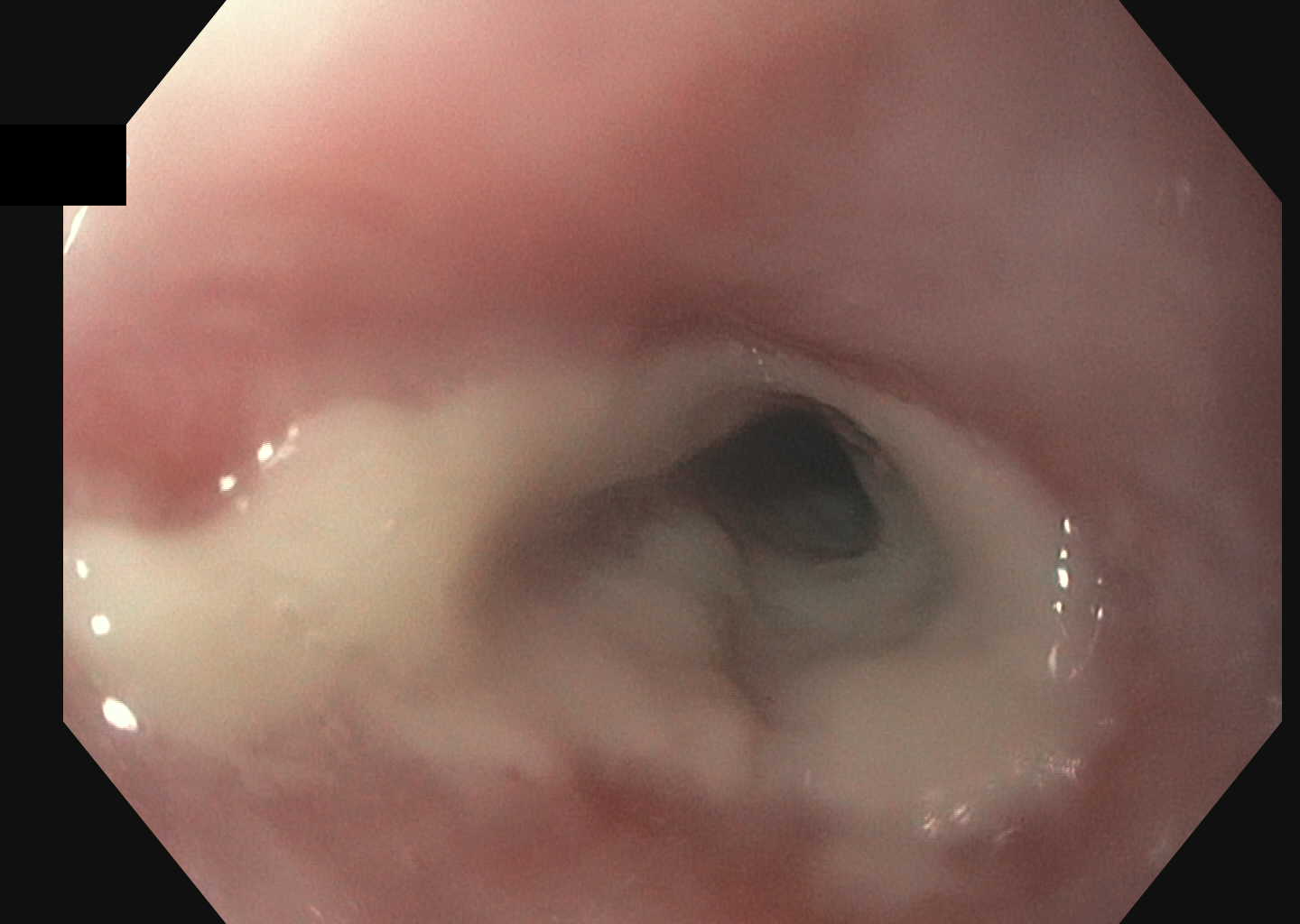


**Supplementary Figure 4:** Endoscopy at 4 months with persistent stricture


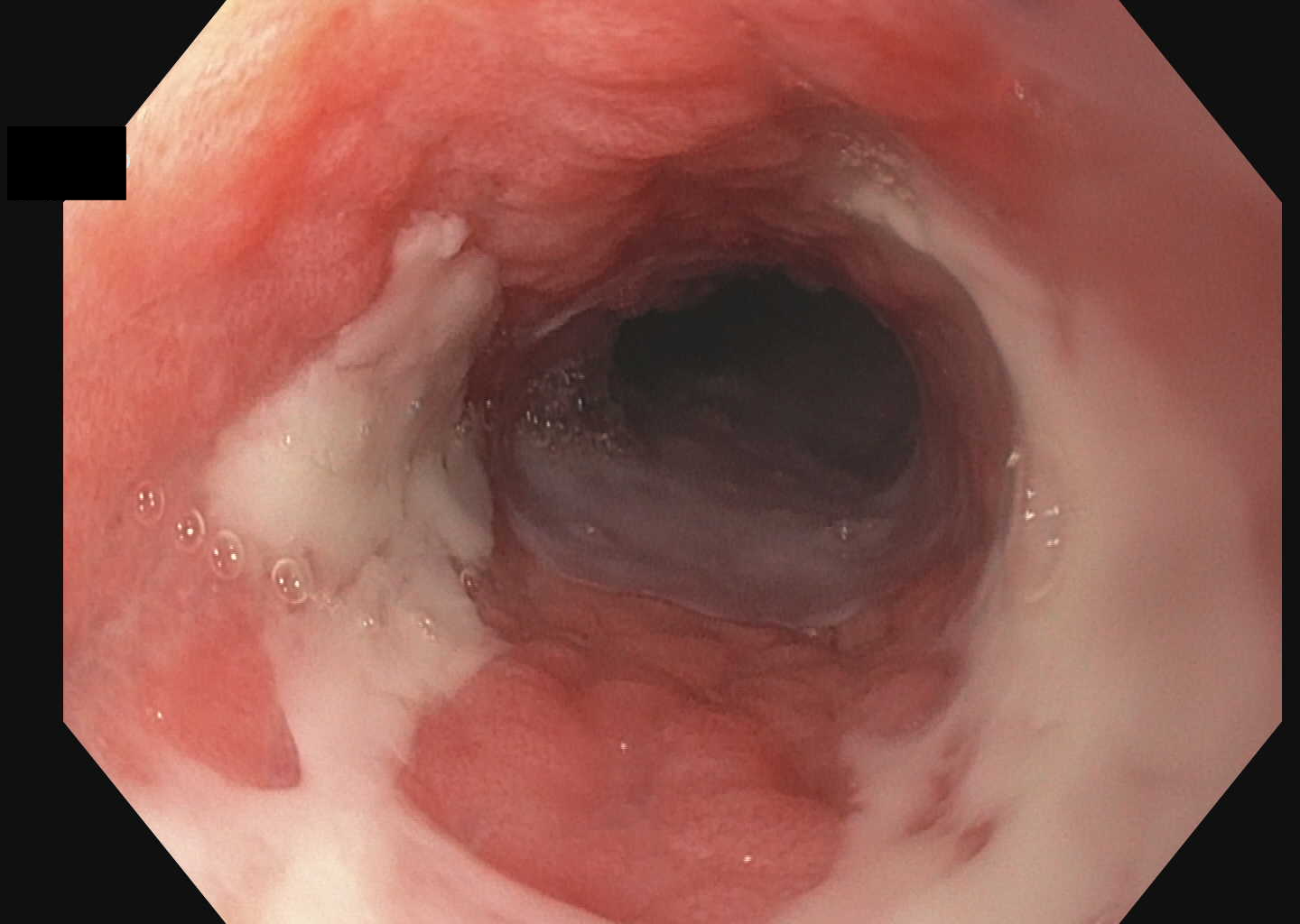


**Supplementary Figure 5:** Endoscopy at 6 months with evidence of esophagitis and nodular-appearing esophageal mucosa
